# Sustainable alginate lyases catalyzed degradation of bio-based carbohydrates

**DOI:** 10.3389/fchem.2022.1008010

**Published:** 2022-09-08

**Authors:** Zhiguo Zheng, Ali Dai, Yonggui Liu, Tingting Li

**Affiliations:** State Key Laboratory Breeding Base of Green Pesticide and Agricultural Bioengineering, Key Laboratory of Green Pesticide and Agricultural Bioengineering, Ministry of Education, Guizhou University, Guiyang, China

**Keywords:** alginate lyase, Brown algae, AOS, agriculture, biological activity

## Abstract

Alginate is a water-soluble and acidic polysaccharide derived from the cell wall and intercellular substance of brown algae. It is widely distributed in brown algae, such as *Laminaria*, *Sargassum,* and *Macrocystis*, etc. Alginate lyase can catalytically degrade alginate in a *β*-eliminating manner, and its degradation product-alginate oligosaccharide (AOS) has been widely used in agriculture, medicine, cosmetics and other fields due to its wide range of biological activities. This article is mainly to make a brief introduction to the classification, source and application of alginate lyase. We hope this minireview can provide some inspirations for its development and utilization.

## Introduction

Alginate is the most abundant linear polysaccharide in brown algae (about 40% of dry weight). It is composed of two uronic acid monomers, *β*-d-mannuronic acid and C5-epimer *α*-l-guluronic acid, through *α*/*β*-1,4 glycosidic bonds in different combinations of poly-guluronic acid (poly-G), poly-mannuronic acid (poly-M), and hybrid fragments of random polymerization of G and M (poly-GM). (Gacesa, 1992; Lee and Mooney, 2012; Kurakake et al., 2017).

At present, the preparation of algal oligosaccharides mainly adopts three kinds of degradation methods: chemical, physical and biological. Chemical methods include acid hydrolysis, alkaline hydrolysis and oxidative degradation, and physical methods mainly include hydrothermal method, ultrasonic method and radiation method, but these two methods have many drawbacks, which are not conducive to large-scale production. Biological methods mainly use microbial fermentation or the action of enzymes to degrade the prepared enzymes. It has the advantages of mild conditions, easy control, and strong product specificity, which has attracted people’s attention, it may be an important direction of industrial production. (Guo et al., 2016).

## Introduction and application of alginate lyase

### Classification of alginate lyases

**FIGURE 1:**
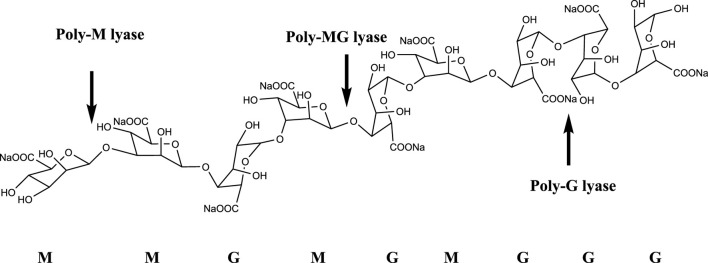
Degradation mode and site of alginate lyase.

The alginate oligosaccharide produced by the enzymatic hydrolysis of alginate lyase has an unsaturated double bond between the C4 and C5 positions of the uronic acid unit at the non-reducing end, and it has a characteristic absorption peak at a specific wavelength of 235 nm. Alginate lyases can be divided into three groups based on substrate specificity, namely poly-G lyase (EC: 4.2.2.11), poly-M lyase (EC: 4.2.2.3), and displays both poly-G and poly-M lyase (Figure 1). In terms of the mode of action, alginate lyase can be divided into endonuclease and exonuclease (Wong et al., 2000). Endonuclease cleaves glycosidic bonds in algin and releases unsaturated oligosaccharides (disaccharides, trisaccharides and tetrasaccharides, etc.), while exonuclease can further degrade oligosaccharides into oligosaccharides. Monomer (Kim et al., 2012; Jagtap et al., 2014). Based on the amino acid sequence alignment, alginate lyases can be classified into different polysaccharide lyase (PL) families including PL5, PL6, PL7, PL8, PL14, PL15, PL17, PL18, PL31, PL32, PL34, PL36, PL39, and PL41 families, which are listed in the Carbohydrate-Active enzymes (CAZy) database (http://www.cazy.org/) (Barzkar et al., 2022).

### Source of alginate lyase

The sources of alginate lyase are extensive, and it has been reported that the production of alginate lyase mainly comes from marine algae, marine mollusks and microorganisms (including bacteria, fungi and some viruses). Among them, there are the most reports on the source of microorganisms, including (*Pseudoalteromonas* sp.) (Ma et al., 2008) (*Vibrio* sp.), (Kawamoto et al., 2006) (*Flavobacterium* sp.) (An et al., 2009) (*Paenibacillus* sp.) (Zhu et al., 2020; Huang et al., 2022).

### Application of alginate lyase

Alginate lyase is an important marine biological enzyme. AOS has various biological activities due to the differences in degradation mode, G content of degradation products (G/M ratio), molecular weight and spatial conformation. As an excellent natural antioxidant, alginate oligosaccharide has great application potential in the fields of human, animals and plants health (Yokose et al., 2009; Tondervik et al., 2014; Saberi Riseh, 2022). It can promote growth, improve stress resistance, increase yield, and inhibit fungal growth. With the implementation of the national strategy of regulating the use of chemical fertilizers and pesticides, it may become an environment-friendly bio-fertilizer and bio-pesticide in the future.

Alginate lyase is not only widely used in agriculture, but also in the fields of medicine and food. It can be used as a growth promoter for therapeutics such as antioxidants and tumor suppressors (Tusi et al., 2011; Hu et al., 2004), and can also induce cytokine production, regulate blood sugar and lipids (Iwamoto et al., 2005), which are widely used in the food and pharmaceutical industries. For example, *Pseudomonas aeruginosa* is one of the main pathogens of many chronic infectious diseases, such as chronic lung infection and urinary tract infection (Jain and Ohman, 2005). The study found that algin is an important component of *Pseudomonas aeruginosa* biofilm, and Albrecht used lyase as an adjunct therapeutic agent together with antibiotics, which made the antibiotics come into direct contact with pathogenic bacteria to achieve the therapeutic effect (Albrecht and Schiller, 2005). This shows that the enzyme has great potential in the bactericidal application of biofilm pathogens.

Due to fuel consumption, researchers have begun to pay attention to the production of biofuel. At present, although chemical catalysis has high efficiency, its high cost, complex synthesis process, and high energy consumption under synthetic conditions limit its industrial application to a certain extent (Pan et al., 2022a; Pan et al., 2022b). Seaweed is considered an ideal source for bioethanol production due to its advantages of not occupying arable land and being non-polluting. According to reports, Wargacki et al. (2012) transformed *Escherichia coli* to establish a system for directly fermenting brown algae to produce ethanol. However, *Escherichia coli* has insufficient tolerance to ethanol, making it impossible for large-scale production. Sasaki et al. (2018) developed a co-cultivation platform for bioethanol production from brown algae, consisting of engineered yeast AM1 and CDY strains, which produced 2.1 g/L of ethanol when the brown algae *Ecklonia kurome* was used as the sole carbon source. This research has made significant progress in the biotechnology of brown algae to bioethanol, but it is still insufficient for industrial production. Studies have shown that the synergistic effect of multiple microorganisms on the ethanol fermentation system of macroalgae will be the trend of future research.

## Conclusion and outlook

Alginate lyase has attracted the attention of researchers because of its unique properties and has great potential for application in various fields. At present, the related research on alginate lyase mainly focuses on the screening of strains, the mining of genes, and the analysis of the degradation substrate and product structure. With the continuous advancement of science and technology, people have gradually deepened the research on the analysis of alginate lyase protein crystal analysis, the catalytic mechanism of the active center and the transformation strategy. This will likely improve the problems of low enzyme-producing strains, low tolerance, and unstable properties in the large-scale industrial production process, and has great application potential in agricultural protection, biofuel production, and environmental protection.
